# Association between Endometriosis and the Risk of Ovarian, Endometrial, Cervical, and Breast Cancer: A Population-Based Study from the U.S. National Inpatient Sample 2016–2019

**DOI:** 10.3390/curroncol31010032

**Published:** 2024-01-13

**Authors:** Ismail Abdulrahman Al-Badawi, Ahmed Abu-Zaid, Osama Alomar, Mohannad Alsabban, Saud Owaimer Alsehaimi, Saad M. S. Alqarni, Safa Nasser Alabdrabalamir, Saeed Baradwan, Maha Al Baalharith, Amal A. AlOdaini, Saleh A. K. Saleh, Heba M. Adly, Ibtihal Abdulaziz Bukhari, Hany Salem

**Affiliations:** 1 Department of Obstetrics and Gynecology, King Faisal Specialist Hospital and Research Center, Riyadh 11211, Saudi Arabia; 2 College of Medicine, Alfaisal University, Riyadh 11533, Saudi Arabia; 3 Department of Obstetrics and Gynecology, King Faisal Armed Forces Hospital, Khamis Mushait 62413, Saudi Arabia; 4 Department of Obstetrics and Gynecology, King Faisal Specialist Hospital and Research Center, Jeddah 23431, Saudi Arabia; 5 Department of Obstetrics and Gynecology, King Abdulaziz Medical City, Riyadh 11426, Saudi Arabia; 6 Department of Pathology, King Fahd University Hospital, College of Medicine, Imam Abdulrahman Bin Faisal University, Dammam 31441, Saudi Arabia; 7 Department of Biochemistry, Faculty of Medicine, Umm Al-Qura University, Makkah 21955, Saudi Arabia; 8 Oncology Diagnostic Unit, Faculty of Medicine, Ain Shams University, Cairo 11435, Egypt; 9 Department of Community Medicine and Pilgrims Healthcare, Faculty of Medicine, Umm Al-Qura University, Makkah 21955, Saudi Arabia; 10 Department of Obstetrics and Gynecology, College of Medicine, Princess Nourah Bint Abdulrahman University, Riyadh 11564, Saudi Arabia

**Keywords:** endometriosis, breast cancer, cervical cancer, ovarian cancer, endometrial cancer, national inpatient sample

## Abstract

Objective: We investigated the potential relationship between endometriosis and risk of ovarian, endometrial, cervical, and breast cancers using the National Inpatient Sample (NIS) database. Methods: We utilized the International Classification of Diseases (ICD-10) system to identify relevant codes from the NIS database (2016–2019). Univariate and multivariate regression analyses (adjusted for age, race, hospital region, hospital teaching status, income Zip score, smoking, alcohol use, and hormonal replacement therapy) were conducted to evaluate the association between endometriosis and gynecologic cancers and summarized as odds ratios (ORs) with 95% confidence intervals (CIs). Results: In the examined dataset, there were 1164 and 225,323 gynecologic cancer patients with and without endometriosis, respectively. Univariate analysis showed endometriosis was significantly associated with a higher risk of ovarian (OR = 3.42, 95% CI: 3.05–3.84, *p* < 0.001) and endometrial (OR = 3.35, 95% CI: 2.97–3.79, *p* < 0.001) cancers. There was no significant association between endometriosis and cervical cancer (OR = 1.05, 95% CI: 0.85–1.28, *p* = 0.663). Interestingly, endometriosis was significantly associated with a low risk of breast cancer (OR = 0.12, 95% CI: 0.10–0.17, *p* < 0.001). Multivariate analysis after Bonferroni correction (*p* < 0.006) showed that endometriosis was significantly associated with a high risk of ovarian (adjusted OR = 3.34, 95% CI: 2.97–3.75, *p* < 0.001) and endometrial (adjusted OR = 3.61, 95% CI: 3.12–4.08, *p* < 0.001) cancers. Conversely, there was no significant association between endometriosis and cervical cancer (OR = 0.80, 95% CI: 0.65–0.99, *p* = 0.036). Conclusions: Patients with endometriosis exhibited unique gynecologic cancer risk profiles, with higher risks for ovarian and endometrial cancers, and no significant risk for cervical cancer. The observed connection between endometriosis and a reduced risk of breast cancer remains a perplexing phenomenon, which cannot be put into context to date.

## 1. Introduction

Endometriosis is a chronic gynecologic disorder manifested by the presence of lesions resembling endometrial tissue located beyond the confines of the uterus [1]. From an epidemiological perspective, endometriosis impacts approximately 5–10% of women during their reproductive years and is linked to a wide array of manifestations, such as chronic pelvic pain, dyspareunia, dysmenorrhea, and infertility [2].

While classified as a benign condition, endometriosis has been shown to share certain characteristics with malignant tumors, such as chronic inflammation, resistance to apoptosis, neoangiogenesis, tissue invasion, and the potential to spread to local and distant sites [3,4,5,6]. Genomic data from cancer studies have unveiled genetic connections between endometriosis and various female cancers, namely breast, cervical, endometrial, and ovarian cancers [7,8]. Moreover, accumulating research from various high-hierarchy systematic review and meta-analysis reports of case-control and cohort studies has documented correlational relationships between endometriosis and certain gynecologic cancers [9,10,11]. Despite conflicting results, these reports have ultimately depicted that endometriosis is positively correlated with a high risk of ovarian, endometrial, and breast cancers, whereas endometriosis is negatively correlated with a low risk of cervical cancer [9,10,11]. Nonetheless, these prior meta-analyses should be interpreted with caution secondary to inherent methodology-related limitations, such as ascertainment and temporality of endometriosis diagnosis, population sampling, study quality, publication bias, and unadjusted confounding.

Population-based national databases play a crucial role in enhancing our comprehension of correlational investigations. Additionally, they help in overcoming the scarcity of available clinical data and lack of robust regression analyses. The National Inpatient Sample (NIS) is one of the most extensive healthcare databases in the United States [12]. It empowers researchers to investigate patterns, correlations, and outcomes associated with various medical conditions, including endometriosis and female gynecologic cancers, within a diverse and representative patient population. Population-level (national-based) data on the relationship between endometriosis and gynecologic cancers are sparse.

Herein, we present the first-ever comprehensive report from the NIS database examining the association between endometriosis and occurrence of select gynecologic cancers (ovarian, endometrial, cervical, and breast). The utilization of the NIS database offers insights into the epidemiology of endometriosis and its relationship with gynecologic cancers and informs clinical practice guidelines for managing this specific cohort of women.

## 2. Methods

Our study sought to comprehensively investigate the relationship between endometriosis and select gynecologic cancers, encompassing ovarian, endometrial, cervical, and breast cancers. Our dataset consisted of a robust collection of records from the NIS database spanning a four-year period, specifically from 2016 to 2019. We applied the International Classification of Diseases, Tenth Revision (ICD-10) coding system to identify relevant codes for our targeted variables (Supplementary Table S1). We conducted a rigorous screening process, excluding records with missing values or those that did not align with our inclusion criteria. We did not exclude patients with missing values in body mass index (BMI) due to the high percentages of absent values. The missing values, whenever applicable, were reported accordingly.

To provide a comprehensive overview of our dataset, we employed descriptive statistics to summarize the fundamental characteristics of our key variables. The chi-squared (χ²) test was employed to assess disparities in baseline characteristics, particularly categorical variables, between patients with endometriosis and those without it. Subsequently, we conducted a series of univariate and multivariate regression analyses to comprehend and quantify the potential relationships between endometriosis and the frequency of female gynecologic cancers. In this analysis, we carefully considered several factors, including age, race, hospital region, smoking status, alcohol use, hormone replacement therapy, hospital teaching status, and income Zip score, as possible confounders. Results were reported as odds ratios (ORs) with 95% confidence intervals (CIs). To avoid a possible increase in type 1 errors during the use of multiple regression models, adjustment using Bonferroni correction was used by dividing the alpha error value (0.05) over the number of tested covariates (n = 8). The statistically significant *p*-value after Bonferroni correction was set at <0.006.

Ethical approval was not required for our study, as we exclusively utilized publicly available, de-identified data from the NIS database.

## 3. Results

Table 1 summarizes the baseline characteristics of patients with (*n* = 1164) and without (*n* = 225,323) endometriosis, according to various demographic and clinical parameters. Notably, some patients with (*n* = 45) and without (*n* = 2486) endometriosis had two or more gynecologic cancers. Overall, patients with endometriosis had significantly higher rates of ovarian (46.56% vs. 20.28%, *p* < 0.05) and endometrial (34.27% vs. 13.46%, *p* < 0.05) cancers compared to patients without endometriosis. Conversely, patients with endometriosis had significantly lower rates of breast cancer compared to patients without endometriosis (14.34% vs. 59.04%, *p* < 0.05). There was no difference between patients with and without endometriosis regarding the incidence rate of cervical cancer (8.68% vs. 8.32%, *p* > 0.05).

Table 2 summarizes the univariate logistic regression analysis for the association between endometriosis and gynecologic cancers. We found that endometriosis was significantly associated with a higher risk of ovarian (OR = 3.42, 95% CI: 3.05–3.84, *p* < 0.001) and endometrial (OR = 3.35, 95% CI: 2.97–3.79, *p* < 0.001) cancers. There was no significant association between endometriosis and cervical cancer (OR = 1.05, 95% CI: 0.85–1.28, *p* = 0.663). Interestingly, endometriosis was significantly associated with a lower risk of breast cancer (OR = 0.12, 95% CI: 0.10–0.17, *p* < 0.001).

Table 3 summarizes the multivariate logistic regression analysis for the association between endometriosis and gynecologic cancers after adjusting for various confounding factors. After applying the Bonferroni correction for multiple testing, the results revealed that endometriosis was significantly associated with a higher risk of ovarian (OR = 3.34, 95% CI: 2.97–3.75, *p* < 0.001) and endometrial (OR = 3.61, 95% CI: 3.12–4.08, *p* < 0.001) cancers. There was no significant association between endometriosis and cervical cancer (OR = 0.80, 95% CI: 0.65–0.99, *p* = 0.036). Surprisingly, endometriosis was significantly associated with a lower risk of breast cancer (OR = 0.12, 95% CI: 0.11–0.15, *p* < 0.001).

## 4. Discussion

The present study analyzed data from the NIS database from 2016 to 2019, examining the relationship between endometriosis and occurrence of gynecologic cancers. The univariate analysis revealed that endometriosis significantly increases the risks of ovarian and endometrial cancers. The multivariate analysis, after adjusting for various confounding factors and applying Bonferroni correction, also depicted that endometriosis is a significant risk factor for ovarian and endometrial cancers, whereas endometriosis has no significant association with the development of cervical cancer. The association between endometriosis and a low risk of breast cancer remains an odd observation that has yet to be fathomed and validated. This comprehensive report provides valuable insights into the potential relationships between endometriosis and gynecologic cancers.

From an epidemiological standpoint, approximately 5–10% of women in their reproductive years are affected by endometriosis [2]. In our present analysis, the prevalence of endometriosis was <1%, which does not meet the assumption that roughly 10% of women either suffer from endometriosis or have a history of endometriosis. This obvious gap can be ascribed to several inherent limitations of the NIS database. One such limitation is that the sampled population was obtained from only hospital discharges; outpatient encounters and observation-only stays were excluded. Therefore, the NIS database may not fully represent the entire population.

In the present study, the observed connection between endometriosis and a reduced risk of breast cancer remains a perplexing phenomenon, which cannot be put into context to date. A systemic, data-related, methodological error is very unlikely; however, it cannot be excluded completely. The higher incidence of endometriosis diagnoses in patients with pelvic cancers, as opposed to breast cancer, may be partially attributed to the way patient histories are recorded. For instance, a pelvic surgeon might show greater interest in the history of endometriosis in a patient due to potential surgical complexities, potentially resulting in underreporting among breast cancer patients. Additionally, it is plausible that patients with pelvic cancers are more frequently referred for pelvic imaging (for example, pelvic magnetic resonance imaging) compared to those with breast cancer, leading to a higher likelihood of endometriosis diagnoses in the pelvic cancer population. Also, in our analysis, breast cancer diagnosis was most predominant among the patients without endometriosis. Accordingly, the relatively small ratio of breast cancer patients with endometriosis to breast cancer patients without endometriosis could have somehow indirectly influenced the association finding. Collectively, the data on connections between endometriosis and breast cancer are not consistent and the findings should be cautiously interpreted until additional high-quality evidence is generated and validated.

Several large-scale systematic reviews and meta-analysis narratives of case–control and cohort studies have investigated the link between endometriosis and gynecologic cancers [9,10,11]. Among them, Kvaskoff and colleagues conducted the most recent and methodologically robust meta-analysis analyzing the correlation between endometriosis and numerous cancers [10]. Consistent with our multivariate analysis results, endometriosis was significantly associated with a high risk of ovarian cancer (n = 24 studies, summary relative risk = 1.93, 95% CI: 1.68, 2.22). Notably, in a pooled analysis of 13 case–control studies, endometriosis has been depicted as associated with an increased risk of certain histologic types of ovarian cancer, notably clear cell (3.05-fold), low-grade serous (2.11-fold), and endometrioid (2.04-fold) ovarian cancers [13]. Dissimilar to our multivariate results, the correlation between endometriosis and a high risk of endometrial cancer produced a statistically insignificant outcome (n = 17 studies, summary relative risk = 1.23, 95% CI: 0.97–1.57) [10]. Additionally, also contradictory to our multivariate results, the link between endometriosis and breast cancer depicted no statistical significance (n = 20 studies, summary relative risk = 1.04, 95% CI: 1.00–1.09) [10]. All in all, despite the comprehensive analyses, the authors of the meta-analysis pinpointed the need to interpret their conclusions with caution. This is because the meta-analyzed studies had several unavoidable methodology-related shortcomings, such as high between-study heterogeneity, critical risk of bias, population selection, temporality of endometriosis diagnosis prior to cancer development, and uncontrolled confounding.

Bhyan and colleagues carried out an integrated bioinformatic analysis and uncovered compelling indications of a genetic connection between endometriosis and gynecologic cancers, including endometrial, ovarian, cervical, and breast [7]. Leveraging data from next-generation sequencing, the authors amassed an extensive listing of genes associated with endometriosis. Functional analysis substantiated that 39 of these genes played key roles in tumor initiation and cancer progression, with 2 specific genes (ESR1 and PGR) being shared across the gynecologic cancers. Mutational analysis further revealed that eight endometriosis-related genes exhibited a heightened frequency of alterations in gynecologic cancer patients. Collectively, the authors concluded that the identification of shared genetic mechanisms between endometriosis and gynecologic cancers opens avenues for future disease management and treatment, emphasizing the potential for early diagnosis to enhance therapeutic interventions [7]. Indeed, several lines of investigation highlighted the potential malignancy-associated features of endometriosis in terms of recalcitrance to programmed cell death, long-lasting inflammation, intensified vascularization, tissue destruction, and capacity to travel to distant foci [3,4,5,6]. Despite the suggested association between endometriosis and cervical cancer based on genomic assays [7], it should be noted that cervical cancer is de facto, to a larger degree, a sexually transmitted disease caused by human papilloma virus (HPV) infection [14]. Unfortunately, in our analysis, we could not control for the potential confounding factor of HPV infection during analysis of the association between endometriosis and cervical cancer.

Assessing cancer risk in women with endometriosis holds significant public health implications for patients, clinicians, and scientists. The established association between endometriosis and cancer understandably instills concern among women grappling with endometriosis. It is our duty to provide accurate information to patients regarding their long-term cancer risk in comparison to women without endometriosis. Moreover, the revelation of an elevated gynecologic cancer risk among women with endometriosis might prompt clinicians to consider providing more frequent related cancer screenings for this specific group of individuals. Notably, there is currently no specific cancer screening program for individuals with endometriosis. A validated screening method for endometriosis diagnosis using a patient questionnaire exists but is not primarily designed for endometriosis-associated cancer screening [15]. Lastly, considering the current gaps in knowledge pertaining to endometriosis, obtaining a holistic understanding of its association with cancer, through detailed investigation, will contribute to an improved understanding of endometriosis pathophysiology, ultimately improving the diagnostic and therapeutic aspects of endometriosis management.

A key strength of this report is the generalizability of the NIS database to investigate the present research question. To elaborate, the gynecologic cancer diagnosis numbers communicated in the present report are roughly equivalent to the United States numbers provided by the American Cancer Society in 2022, representing about 12% of the overall new cancer diagnoses per annum in the United States [16]. Nonetheless, our study encounters limitations associated with the use of the NIS database. As a sample derived from hospital discharges, the NIS database might not comprehensively reflect the entire population. Depending on administrative/billing data triggers uncertainties regarding coding accuracy, which could result in mistakes or incomplete reporting. The NIS is also deficient in detailed clinic-demographic information, restricting the depth of analysis and resulting in an inability to control for potential confounders. Temporal shifts in coding practices may introduce variability in the data, and the cross-sectional study design hampers the definitive establishment of causation. Despite these constraints, the NIS database continues to be valuable for large-scale population-level studies.

For future directions, prospective research may concentrate on comprehensively dissecting the impact of endometriosis on various clinico-pathological features of ovarian cancer (for example, histologic type and macro-phenotype). Additionally, the substantial ‘protective’ association identified between endometriosis and cervical/breast cancers deserves exploration to ascertain whether it stems from mere luck, biases related to access to care, or if there exists a genuine connection with inherent protective physiological mechanisms in women with endometriosis. If endometriosis plays a causative role in the development of cancer, it logically follows that endometriosis must precede the occurrence of cancer, and this temporality should be established prior to examining any relationship between endometriosis and gynecologic cancers. Lastly, interesting research may explore the correlation between endometriosis and other gynecologic cancers, such as vaginal, vulvar, and fallopian tube tumors.

## 5. Conclusions

Our analysis reveals compelling relationships between endometriosis and gynecologic cancers. Endometriosis significantly increases the risk of ovarian and endometrial cancers. Multivariate analysis, considering various factors, confirms these associations, underscoring their robustness. All in all, these findings emphasize the importance of tailored healthcare management for individuals with endometriosis, considering their unique cancer risk profiles. Notably, the present analysis does not prove causative risks, which would require clinical validation, but observes potentially relevant associations. Further research is warranted to unravel the underlying mechanisms driving this connection.

## Figures and Tables

**Table 1:** The baseline characteristics of analyzed female gynecologic cancer patients with and without endometriosis.

	Patients with Endometriosis				Patients without Endometriosis			
Variable	Ovarian Cancer (n = 542)	Endometrial Cancer (n = 399)	Cervical Cancer (n = 101)	Breast Cancer (n = 167)	Ovarian Cancer (n = 45,705)	Endometrial Cancer (n = 30,325)	Cervical Cancer (n = 18,753)	Breast Cancer (n = 133,026)
**Age** (mean ± standard deviation)	52.30 ± 12.34	60.54 ± 11.36	44.86 ± 12.27	50.68 ± 10.24	53.02 ± 17.31	54.73 ± 15.46	42.21 ± 16.91	53.42 ± 17.23
**Year**								
2016	140 (25.83)	88 (22.06)	25 (24.75)	54 (32.34)	11,272 (24.66)	7390 (24.37)	4598 (24.52)	31,907 (23.99)
2017	136 (25.09)	99 (24.81)	24 (23.76)	33 (19.76)	11,455 (25.06)	7490 (24.70)	4549 (24.26)	32,497 (24.43)
2018	133 (24.54)	104 (26.07)	28 (27.72)	34 (20.36)	11,481 (25.12)	7565 (24.95)	4687 (24.99)	33,577 (25.24)
2019	133 (24.54)	108 (27.07)	24 (23.76)	46 (27.54)	11,497 (25.15)	7880 (25.99)	4919 (26.23)	35,045 (26.34)
**Race**								
White	341 (62.92)	263 (65.91)	71 (70.30)	114 (68.26)	33,036 (72.28)	19,773 (65.20)	10,603 (56.54)	89,335 (67.16)
Black	60 (11.07)	57 (14.29)	13 (12.87)	22 (13.17)	4881 (10.68)	5350 (17.64)	3501 (18.67)	22,517 (16.93)
Hispanic	65 (11.99)	31 (7.77)	11 (10.89)	16 (9.58)	4379 (9.58)	2947 (9.72)	3118 (16.63)	12,121 (9.11)
Asian or Pacific Islander	47 (8.67)	32 (8.02)	3 (2.97)	8 (4.79)	1822 (3.99)	1141 (3.73)	673 (3.59)	4380 (3.29)
Native American	2 (0.37)	2 (0.50)	1 (0.99)	0 (0)	193 (0.42)	122 (0.40)	108 (0.58)	559 (0.42)
Other	27 (4.98)	14 (3.51)	2 (1.98)	7 (4.19)	1394 (3.05)	992 (3.27)	750 (4)	4114 (3.09)
**Primary expected payer**								
Medicare	98 (18.08)	159 (39.85)	11 (10.89)	33 (19.76)	23,630 (51.70)	16,551 (54.58)	5212 (27.79)	69,187 (52.01)
Medicaid	71 (13.10)	37 (9.27)	26 (25.74)	30 (17.96)	4901 (10.72)	3402 (11.22)	6530 (34.82)	16,781 (12.61)
Private insurance	338 (62.36)	186 (46.62)	58 (57.74)	93 (55.69)	15,026 (32.88)	8920 (29.41)	5552 (29.61)	42,261 (31.77)
Self-pay	18 (3.32)	8 (2.01)	3 (2.97)	2 (1.20)	1109 (2.43)	770 (2.54)	923 (4.92)	2028 (1.52)
No charge	4 (0.74)	0 (0.00)	0 (0)	1 (0.60)	120 (0.26)	101 (0.33)	105 (0.56)	258 (0.19)
Other	13 (2.40)	9 (2.26)	3 (2.97)	8 (4.79)	919 (2.01)	581 (1.92)	431 (2.30)	2511 (1.89)
**ZIP income quartile**								
1st–25th	103 (19)	75 (18.80)	24 (23.76)	34 (20.36)	11,210 (24.53)	8532 (28.14)	6922 (36.91)	35,095 (26.38)
26th–50th	121 (22.32)	97 (24.31)	22 (21.78)	31 (18.56)	11,392 (24.93)	7472 (24.64)	4763 (25.40)	32,343 (24.31)
51st–75th	135 (24.91)	99 (24.81)	24 (23.76)	39 (23.35)	11,597 (25.37)	7488 (24.69)	4130 (22.02)	32,873 (24.71)
76th–100th	183 (33.76)	128 (32.08)	31 (30.69)	63 (37.72)	11,506 (25.17)	6833 (22.53)	2938 (15.67)	32,715 (24.59)
**Location/Teaching status of hospital**								
Rural	10 (1.85)	5 (1.25)	3 (2.97)	14 (8.38)	2127 (4.65)	1186 (3.91)	864 (4.61)	9355 (7.03)
Urban nonteaching	45 (8.30)	38 (9.52)	12 (11.88)	27 (16.17)	7397 (16.18)	3981 (13.13)	2833 (15.11)	26,824 (20.16)
Urban teaching	487 (89.85)	356 (89.22)	86 (85.15)	126 (75.45)	36,181 (79.16)	25,158 (82.96)	15,056 (80.29)	96,847 (72.80)
**Bed Size**								
Small	53 (9.78)	38 (9.52)	12 (11.88)	27 (16.17)	6401 (14.01)	3866 (12.75)	2327 (12.41)	25,589 (19.24)
Medium	116 (21.40)	76 (19.05)	29 (28.71)	57 (34.13)	11,967 (26.18)	7550 (24.90)	4627 (24.67)	38,230 (28.74)
Large	373 (68.82)	285 (71.43)	60 (59.41)	83 (49.70)	27,337 (59.81)	18,909 (62.35)	11,799 (62.92)	69,207 (52.03)
**Hospital Region**								
Northeast	137 (25.28)	102 (25.56)	26 (25.74)	42 (25.15)	9241 (20.22)	6743 (22.24)	3237 (17.26)	29,592 (22.25)
Midwest or North Central	83 (15.31)	68 (17.04)	16 (15.84)	26 (15.57)	9626 (21.06)	7064 (23.29)	3483 (18.57)	28,061 (21.09)
South	183 (33.76)	137 (34.34)	33 (32.67)	68 (40.72)	16,915 (37.01)	10,482 (34.57)	8248 (43.98)	50,408 (37.89)
West	139 (25.65)	92 (23.06)	26 (25.74)	31 (18.56)	9923 (21.71)	6036 (19.90)	3785 (20.18)	24,965 (18.77)
**Body Mass Index** (kg/m^2^)								
<20	7 (1.29)	3 (0.75)	3 (2.97)	2 (1.20)	2110 (4.62)	559 (1.84)	1468 (7.83)	4389 (3.30)
20–30	12 (2.21)	5 (1.25)	3 (2.97)	3 (1.80)	3181 (6.96)	1321 (4.36)	1090 (5.81)	6158 (4.63)
30–40	45 (8.30)	49 (12.28)	9 (8.91)	6 (3.59)	3134 (6.86)	3643 (12.01)	1145 (6.11)	9422 (7.08)
>40	35 (6.46)	80 (20.05)	7 (6.93)	6 (3.59)	2405 (5.26)	5439 (17.94)	986 (5.26)	7583 (5.70)
Missing	443 (81.73)	262 (65.66)	79 (78.22)	150 (89.82)	34,875 (76.30)	19,363 (63.85)	14,064 (75)	105,474 (79.29)
**Hormonal replacement therapy**	6 (1.11)	5 (1.25)	0 (0)	2 (1.20)	412 (0.90)	253 (0.83)	84 (0.45)	1086 (0.82)
**Alcohol misuse**	2 (0.37)	0 (0)	3 (2.97)	1 (0.60)	168 (0.37)	75 (0.25)	134 (0.71)	752 (0.57)
**Smoking**	0 (0)	3 (0.75)	0 (0)	1 (0.60)	203 (0.44)	117 (0.39)	191 (1.02)	721 (0.54)
**Died**	2 (0.37)	2 (0.50)	1 (0.99)	3 (1.80)	2128 (4.66)	1071 (3.53)	716 (3.82)	6017 (4.52)

**Table 2:** Univariate regression analysis of the association between endometriosis and risk of ovarian, endometrial, cervical, and breast cancer.

Cancer Type	Univariate Analysis	
	Odds Ratio [95% Confidence Interval]	*p*-Value
Ovarian cancer	3.42 [3.05, 3.84]	<0.001
Endometrial cancer	3.35 [2.97, 3.79]	<0.001
Cervical cancer	1.05 [0.85, 1.28]	0.663
Breast cancer	0.12 [1.10, 1.17]	<0.001

**Table 3:** Multivariate regression analysis of the association between endometriosis and risk of ovarian, endometrial, cervical, and breast cancer.

Cancer Type	Multivariate Analysis	
	Adjusted Odds Ratio [95% Confidence Interval]	*p*-Value
Ovarian cancer	3.34 [2.97, 3.75]	<0.001 *
Endometrial cancer	3.61 [3.19, 4.08]	<0.001 *
Cervical cancer	0.80 [0.65, 0.99]	0.036
Breast cancer	0.12 [0.11, 0.15]	<0.001 *

Multivariate analysis was adjusted for age, race, hospital region, hospital teaching status, income Zip score, smoking, alcohol misuse, and hormonal replacement therapy. * Statistical significance after Bonferroni correction (*p* < 0.006).

## Data Availability

The data that support the findings of this study are available on request from the corresponding author.

## Supplementary Materials

The following supporting information can be downloaded at: https://www.mdpi.com/article/10.3390/curroncol31010032/s1, Table S1: The codes for data analysis and their source.
