# Supplementary material for: Association between Endometriosis and the Risk of Ovarian, Endometrial, Cervical, and Breast Cancer: A Population-Based Study from the U.S. National Inpatient Sample 2016–2019
Source: Curr Oncol. 2024 Jan 13;31(1):472–81. doi: 10.3390/curroncol31010032 (PMC10814716; doi:10.3390/curroncol31010032)
Supplement: Supplementary file 1 [file curroncol-31-00032-s001.zip › curroncol-2778536-supplementary.pdf]

**Supplementary Table 1**. The codes for data analysis and their source.

| Variable | Source | ICD-10 code |
|---|---|---|
| Endometriosis | I10_DX1/40 | N80 |
| Endometrial cancer | I10_DX1/40 | C54 |
| Ovarian cancer | I10_DX1/40 | C56 |
| Cervical cancer | I10_DX1/40 | C53 |
| Breast cancer | I10_DX1/40 | C50 |
| Body mass index | | |
| 19.9 or less | I10_DX1/40 | Z68.1 |
| 20-29 | I10_DX1/40 | Z68.2 |
| 30-39 | I10_DX1/40 | Z68.3 |
| 40 or greater | I10_DX1/40 | Z68.4 |
| Hormone replacement therapy | I10_DX1/40 | Z79.890 |
| Alcohol misuse | I10_DX1/40 | F10.1 |
| Smoking (tobacco use) | I10_DX1/40 | Z72.0 |
| Age | NIS Core | - |
| Primary expected payer | NIS Core | - |
| Race | NIS Core | - |
| Year | NIS Core | - |
| ZIP income quartile | NIS Core | - |
| Hospital bed size | NIS Hospital | - |
| Hospital Region | NIS Hospital | - |
| Location/Teaching status of hospital | NIS Hospital | - |
